# Supplementary material for: Lysosomal Ca2+-mediated TFEB activation modulates mitophagy and functional adaptation of pancreatic β-cells to metabolic stress
Source: Nat Commun. 2022 Mar 14;13:1300. doi: 10.1038/s41467-022-28874-9 (PMC8921223; doi:10.1038/s41467-022-28874-9)
Supplement: Supplementary file 1 — Supplementary Information [file 41467_2022_28874_MOESM1_ESM.pdf]

Supplementary Information

Lysosomal Ca^{2+}-mediated TFEB activation modulates mitophagy and functional adaptation of pancreatic β-cells to metabolic stress

Kihyoun Park, Hyejin Lim, Jinyoung Kim, Yeseong Hwang, Yu Seol Lee, Soo Han Bae, Hyeongseok Kim, Hail Kim, Shin-Wook Kang, Joo Young Kim & Myung-Shik Lee

Correspondence to: mslee0923@yuhs.ac

Supplementary Figure. 1-12

Supplementary Table 1-4

Supplementary Fig. 1

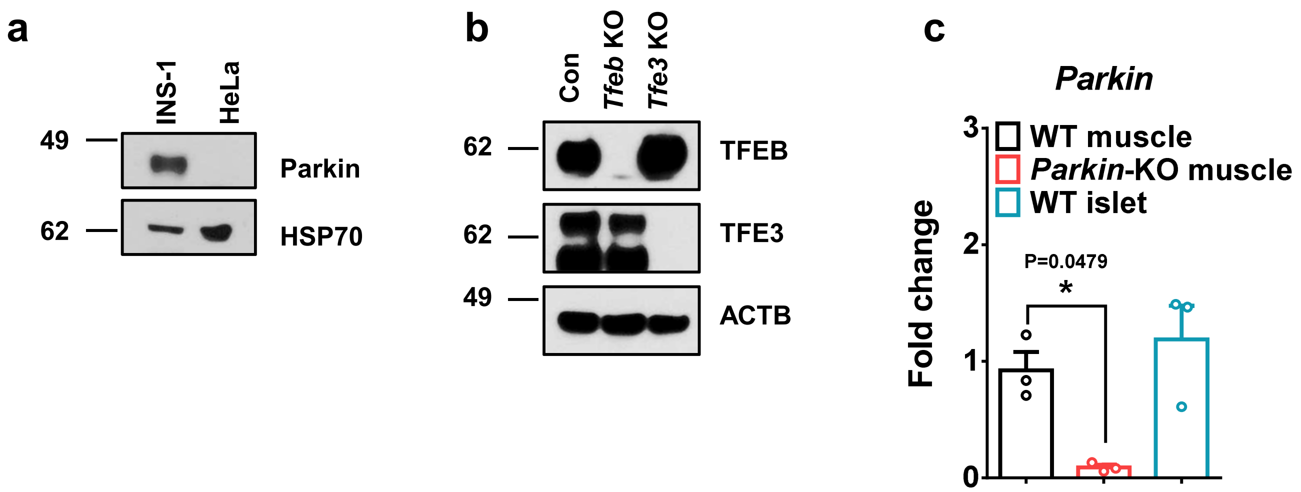

Supplementary Fig. 1. Expression of Parkin in insulinoma cells and islet cells. a Immunoblotting of lysate prepared from INS-1 and HeLa cells was conducted using anti-Parkin Ab. HeLa cells that did not express Parkin[4] were used as controls. **b** Immunoblotting of lysate prepared from Cas9 Control (Con), *Tfeb*-KO and *Tfe3*-KO INS-1 cells using the indicated Abs. **c** mRNA from muscle of *Parkin*-KO or wild-type (WT) mice and pancreatic islets of WT mice were subjected to real-time RT-PCR using specific primers for *Parkin*. (n=3) All data in this figure are the means ± SEM from more than 3 independent experiments. *P* values were determined using one-way ANOVA with Tukey's test.

Supplementary Fig. 2

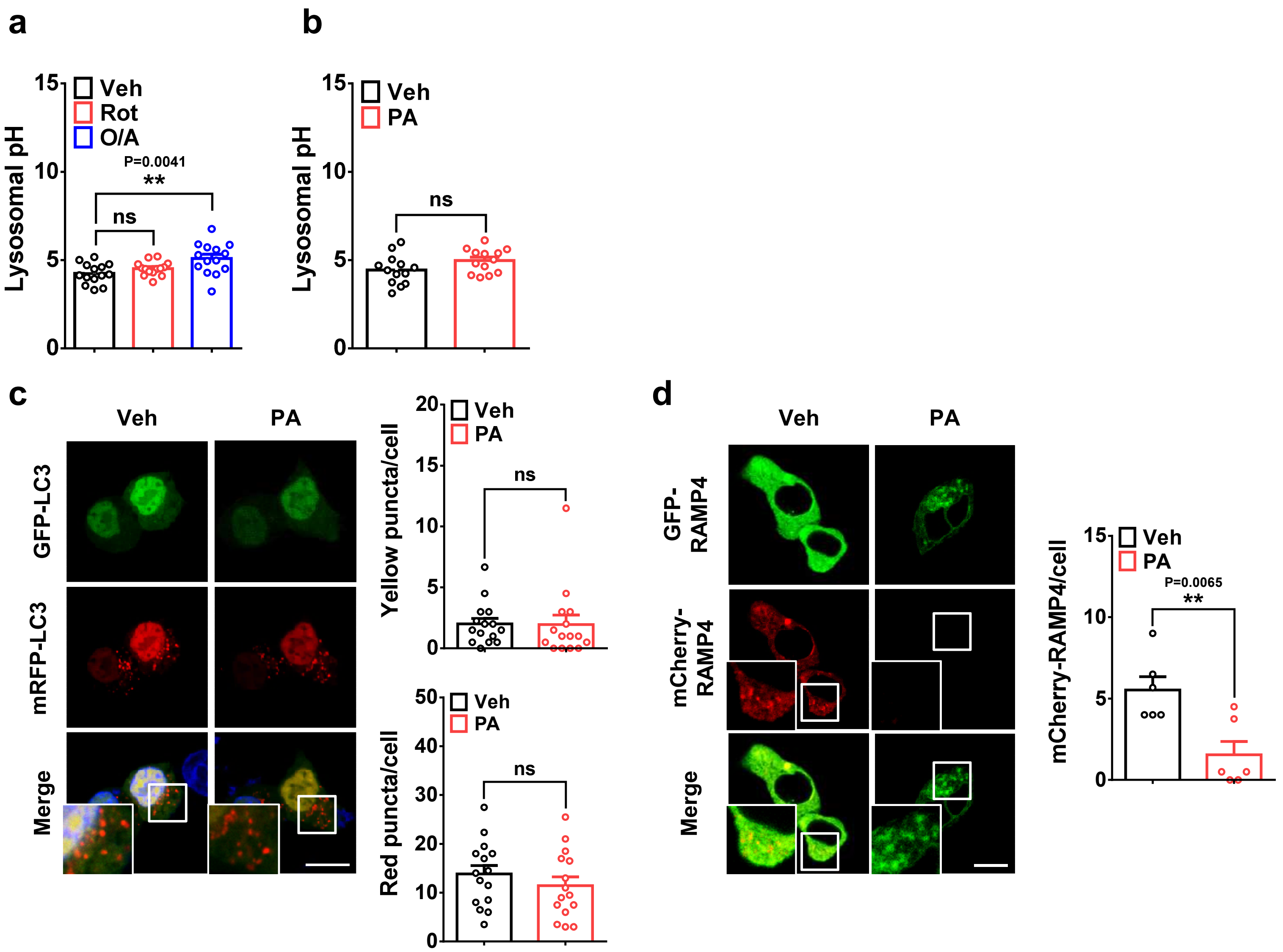

Supplementary Fig. 2. Effect of mitochondrial stressors or PA on autophagic flux and lysosomal pH. a After treatment of INS-1 cells with rotenone or O/A for 1 h, lysosomal pH was determined as described in the Methods. (*n*=14) **b**. After treatment of INS-1 cells with PA for 1 h, lysosomal pH was determined as in (**a**). (*n*=13) **c** INS-1 cells transfected with *mRFP-GFP-LC3* plasmid were treated with PA for 16 h, and the number of red puncta representing autophagolysosome and that of yellow puncta representing autophagosome were counted (right). Representative confocal images are presented (left). (scale bar, 10 µm) (*n*=15) **d** INS-1 cells transfected with *TetOn-mCherry-GFP-RAMP4* and then treated with 4 µg/ml Doxycycline for 24 h were subjected to PA treatment for 16 h. The number of red puncta representing ER-phagic flux was counted (right). Representative confocal images are presented (left). (scale bar, 10 µm) (*n*=6) Cells in the rectangles were magnified. All data in this figure are the means ± SEM from more than 3 independent experiments. *P* values were determined using one-way ANOVA with Tukey's test [**a**] or two-tailed t-test [**b,c,d**]; ns, not significant.

Supplementary Fig. 3

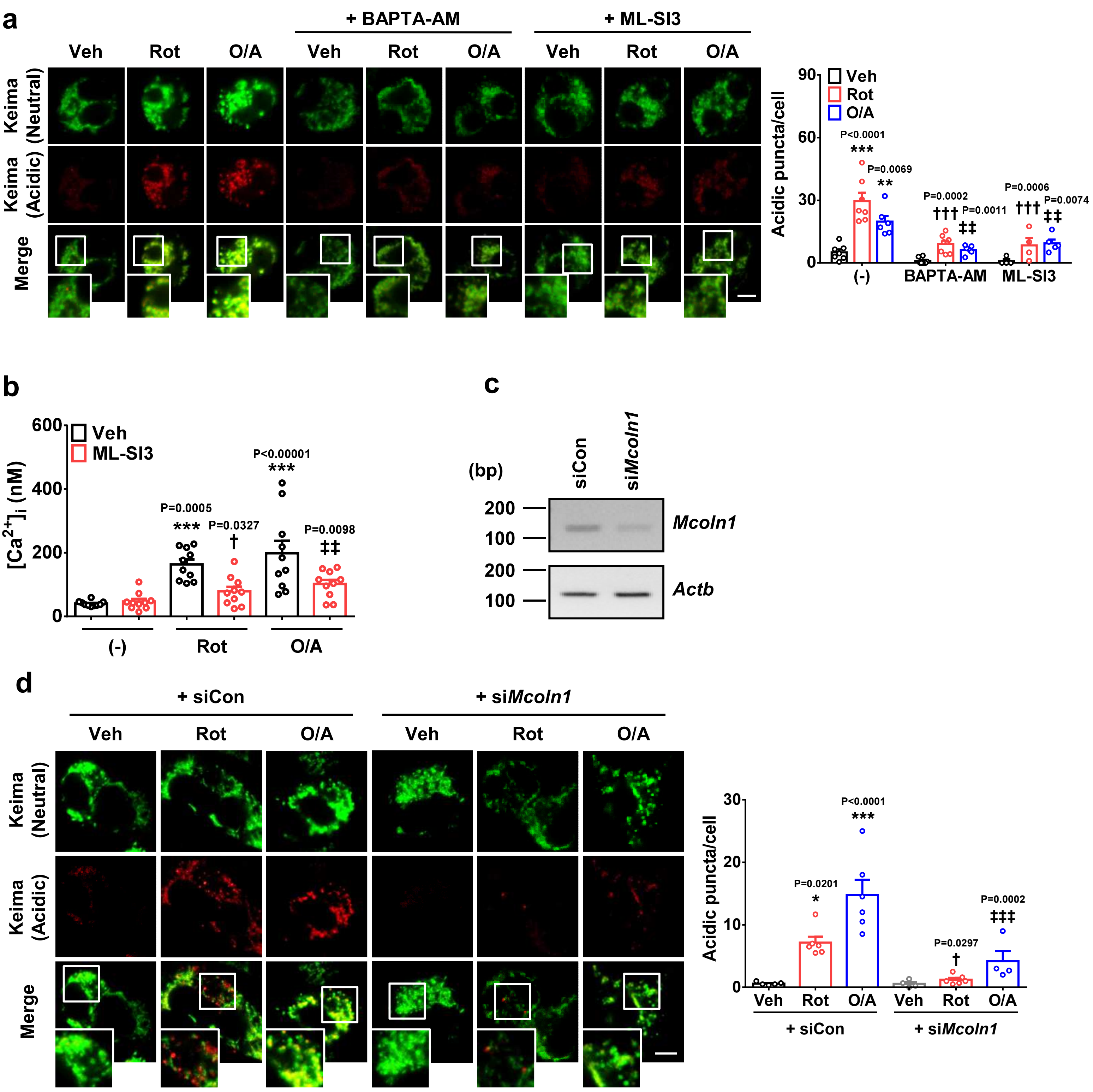

Supplementary Fig. 3. Role of cytosolic Ca^{2+} and TRPML1 channel in mitochondrial stress-induced mitophagy. a INS-1 cells were transfected with *pMito-Keima*, and then treated with rotenone or O/A for 18 h after BAPTA-AM or ML-SI3 pretreatment. Keima fluorescence at neutral pH and in acidic condition was examined by confocal microscopy (left). The number of red puncta indicating mitophagy per cell was counted (right). (scale bar, 5 μm) (*n*=4 for ML-SI3 or ML-SI3+Rot; *n*=5 for ML-SI3+O/A; *n*=6 for O/A or BAPTA-AM+O/A; *n*=7 for Veh or Rot; *n*=8 for BAPTA-AM or BAPTA-AM+Rot) **b** Effect of a TRPML1 channel antagonist on $[Ca^{2+}]_i$. After pretreatment with ML-SI3, an antagonist of TRPML1 channel, for 1 h, INS-1 cells were treated with rotenone or O/A, and $[Ca^{2+}]_i$ was determined by a ratiometric method using Fura-2. (*n*=10) **c** Expression of *Mcoln1* in INS-1 cells transfected with *Mcoln1* siRNA (si*Mcoln1*) was examined by RT-PCR. **d** INS-1 cells transfected with *Mcoln1* siRNA and *pMito-Keima* were treated with rotenone or O/A, and the numbers of red puncta showing occurrence of mitophagy were counted (right). Representative fluorescent images are presented (left). (scale bar, 5 μm) (*n*=4 for si*Mcoln1* or si*Mcoln1*+O/A; *n*=5 for siCon; *n*=6 for siCon+Rot, siCon+O/A or si*Mcoln1*+Rot) (SiCon, control siRNA) Cells in the rectangles were magnified. All data in this figure are the means ± SEM from more than 3 independent experiments. *P* values were determined using one-way ANOVA with Tukey's test. *, compared to cells or control transfectants treated with Veh; †, compared to cells or control transfectants treated with Rot alone; ‡, compared to cells or control transfectants treated with O/A alone.

Supplementary Fig. 4

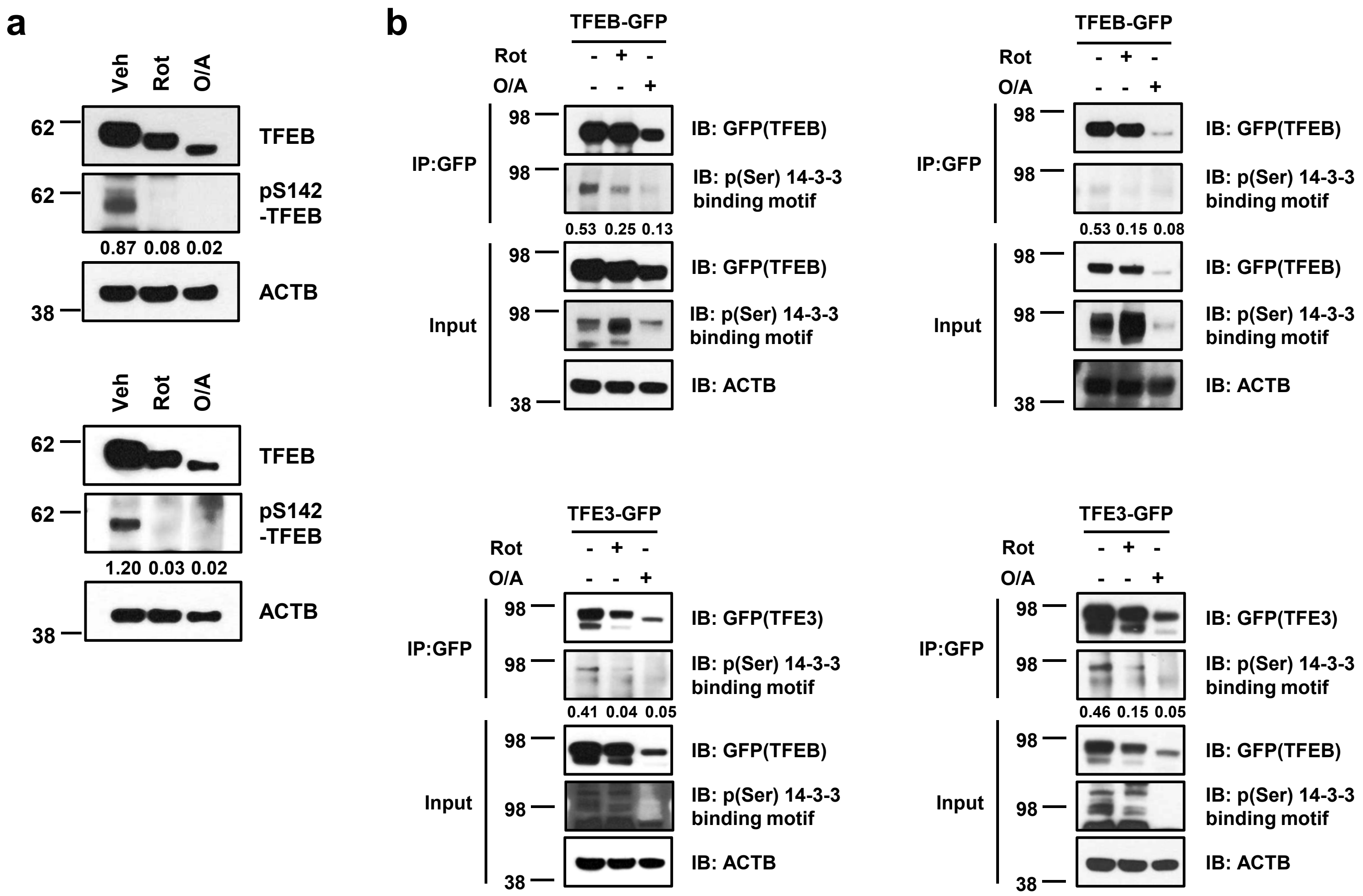

Supplementary Fig. 4. Dephosphorylation of TFEB and TFE3 by mitochondrial stressors. a INS-1 cells were treated with rotenone or O/A for 4 h, and cell extract was subjected to immunoblot analysis (IB) using the indicated Abs. (*n*=2) **b** After lysis of *TFEB-GFP-* (upper; *n*=2) or *TFE3-GFP*-transfected INS-1 cells (lower; *n*=2) treated with rotenone or O/A for 4 h, immunoprecipitation (IP) were conducted using anti-GFP Ab and Protein-G beads. Supernatant was collected by centrifugation after heating the beads in a sample buffer, and then subjected to IB using the indicated Abs. Densitometry of IB bands was performed using ImageJ. Numbers below IB bands indicate the densitometric values normalized to that of the TFEB or TFE3 band.

Supplementary Fig. 5

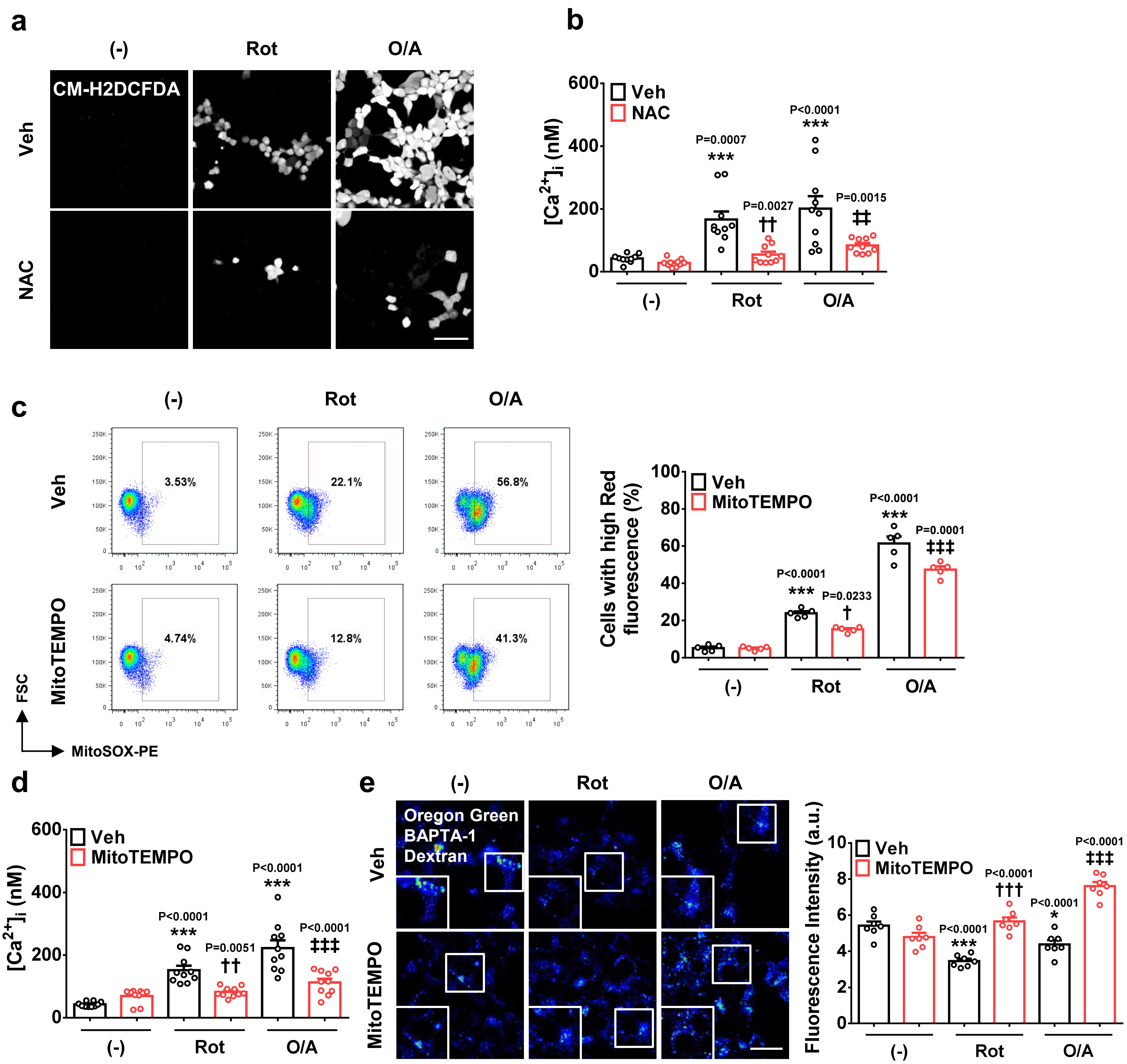

Supplementary Fig. 5. Role of mitochondrial ROS in lysosomal Ca^{2+} efflux. a After rotenone or O/A treatment of INS-1 cells for 1 h with or without NAC pretreatment for 1 h, cellular ROS was determined using CM-H2DCFDA. (scale bar, 50 μm) **b** After rotenone or O/A treatment of INS-1 cells for 1 h with or without NAC pretreatment for 1 h, $[Ca^{2+}]_i$ was determined by a ratiometric method using Fura-2. (*n*=10) **c** After rotenone or O/A treatment of INS-1 cells for 1 h with or without MitoTEMPO pretreatment for 1 h, mitochondrial ROS was determined using MitoSOX (right). Representative scattergrams are presented (left). (*n*=5) **d** After rotenone or O/A treatment of INS-1 cells for 1 h with or without MitoTEMPO pretreatment for 1 h, $[Ca^{2+}]_i$ was determined by a ratiometric method using Fura-2. (*n*=10) **e** $[Ca^{2+}]_{Lys}$ was determined after rotenone or O/A treatment of Oregon Green 488 BAPTA-1 Dextran-loaded INS-1 cells for 1 h with or without MitoTEMPO pretreatment for 1 h (right). Representative fluorescent images are presented (left). (scale bar, 20 μm) (*n*=7) Cells in the rectangles were magnified. All data in this figure are the means ± SEM from more than 3 independent experiments. *P* values were determined using one-way ANOVA with Tukey's test. *, compared to Veh-treated cells; †, compared to cells treated with Rot alone; ‡, compared to cells treated with O/A alone.

Supplementary Fig. 6

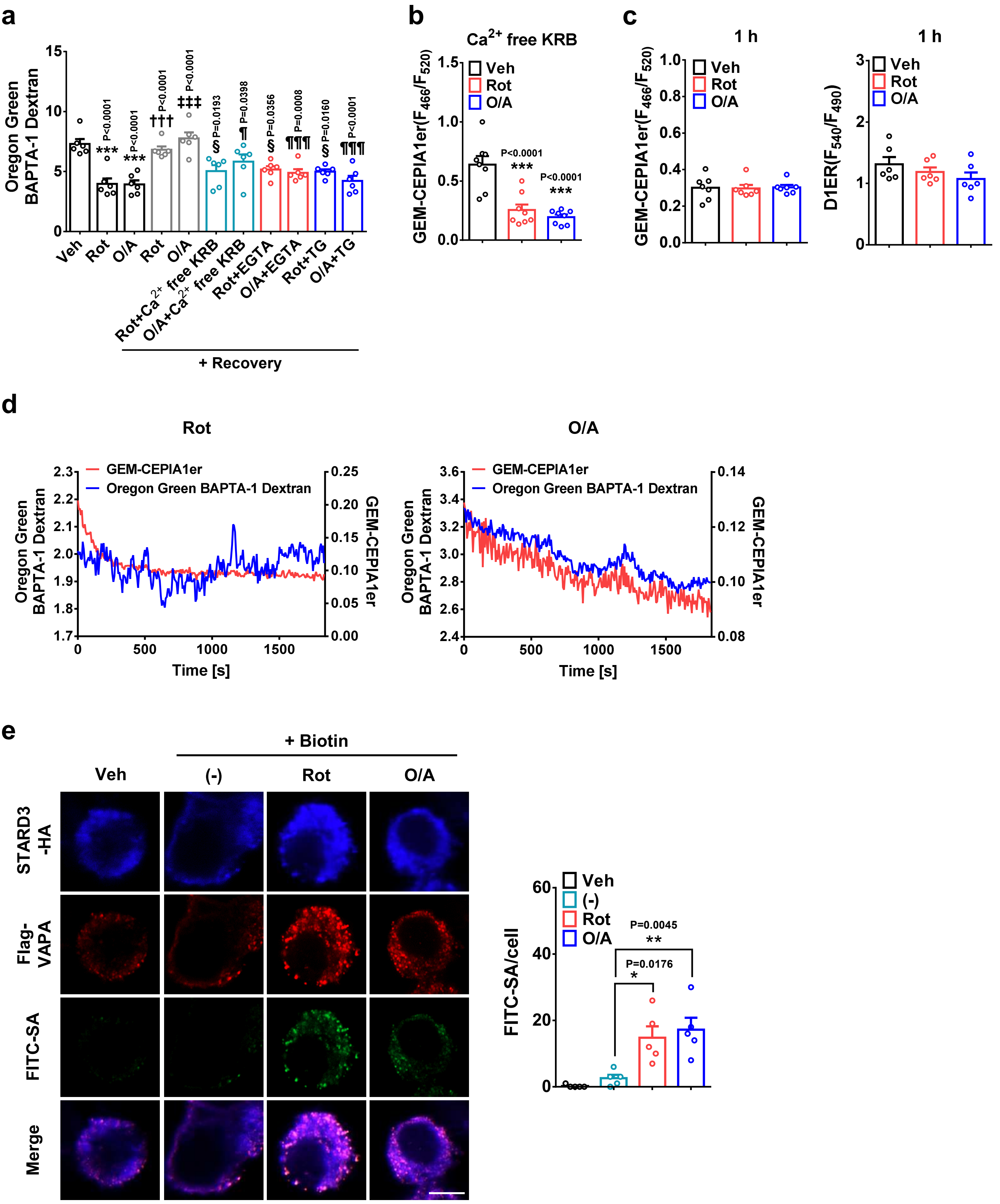

Supplementary Fig. 6. **ER→lysosome Ca^{2+} refilling and ER-lysosome contact during mitophagy.** **a** Oregon Green 488 BAPTA-1 Dextran-labelled INS-1 cells were treated with mitochondrial stressors for 1 h. After removal of stressors for 1 h, recovery of $[Ca^{2+}]_{Lys}$ was determined with or without 2 mM EGTA or 2 μM Thapsigargin and in a Ca^{2+}-free KRB buffer. (*n*=6) **b** $[Ca^{2+}]_{ER}$ was determined after treating *GEM-CEPIA1er*-transfected cells with mitochondrial stressors for 1 h in the absence of extracellular Ca^{2+}. (*n*=8) **c** INS-1 cells transfected with *GEM-CEPIA1er* or a ratiometric FRET-based Cameleon probe *D1ER* were treated with rotenone or O/A for 1 h without removal of extracellular Ca^{2+}. (*n*=7 for GEM-CEPIA1er; *n*=6 for D1ER) **d** Cells transfected with *GEM-CEPIA1er* and labelled with Oregon Green 488 BAPTA-1 Dextran were treated with mitochondrial stressors for 1 h. Recovery of $[Ca^{2+}]_{Lys}$ and changes in $[Ca^{2+}]_{ER}$ were determined simultaneously without removal of mitochondrial stressors (rotenone or O/A) in the absence of extracellular Ca^{2+}. **e** INS-1 cells transfected with Flag-BirA(N-G78)-VAPA and STARD3-BirA(G79-C)-HA as described in the Methods were treated with mitochondrial stressors. Formation of biotin conjugate, thus, the contact between VAPA on ER membrane and STARD3 on lysosomal membrane was examined by FITC-streptavidin (SA) staining and confocal microscopy (left). The number of FITC-SA spots was counted using ImageJ (right). (scale bar, 5 μm) (*n*=5) All data in this figure are the means ± SEM from more than 3 independent experiments. *P* values were determined using one-way ANOVA with Tukey's test. *, compared to Veh-treated cells; †, compared to cells treated with Rot alone; ‡, compared to cells treated with O/A alone; §, compared to Rot-treated cells after recovery; ¶, compared to O/A-treated cells after recovery.

Supplementary Fig. 7

a

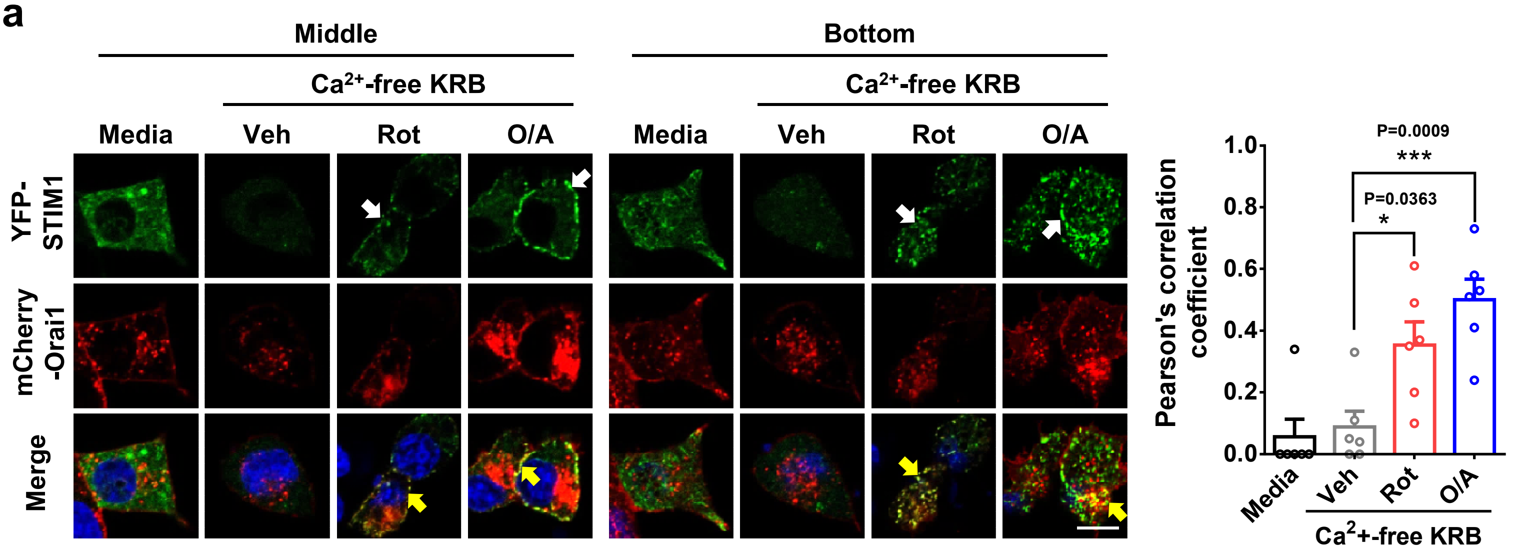

b

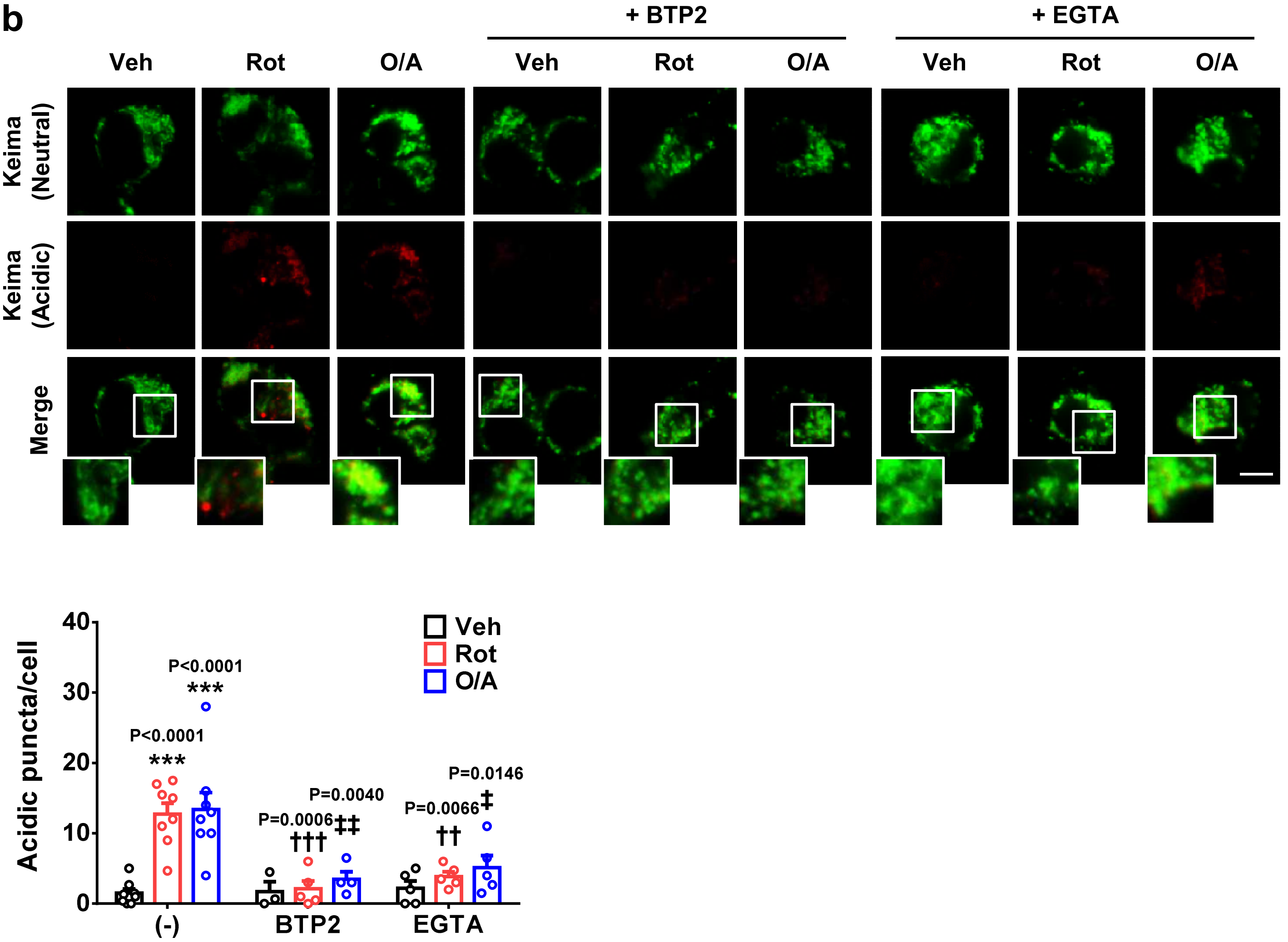

Supplementary Fig. 7. Effect of CRAC and extracellular Ca^{2+} on mitophagy. a INS-1 cells transfected with *YFP-STIM1* and *mCherry-Orai1* were treated with mitochondrial stressors in a Ca^{2+}-free KRB buffer for 30 min, and STIM1 oligomerization (white arrow) and colocalization with Orai1 (yellow arrow) were determined by confocal microscopy at the middle and bottom levels (left). Pearson's correlation coefficients were calculated as measures of colocalization (right). (scale bar, 10 μm) (*n*=6) **b** *pMito-Keima*-transfected INS-1 cells were treated with rotenone or O/A for 18 h in the presence or absence of BTP2 inhibiting extracellular Ca^{2+} influx or EGTA chelating extracellular Ca^{2+}. The number of red acidic puncta, thus mitophagy, in transfected cells was determined (lower). Representative fluorescent images are presented (upper). (scale bar, 5 μm) (*n*=3 for BTP2; *n*=4 for BTP2+O/A; *n*=5 for BTP2+Rot, EGTA, EGTA+Rot or EGTA+O/A; *n*=8 for Veh, Rot or O/A) Cells in the rectangles were magnified. All data in this figure are the means ± SEM from more than 3 independent experiments. *P* values were determined using one-way ANOVA with Tukey's test. *, compared to Veh-treated cells; †, compared to cells treated with Rot alone; ‡, compared to cells treated with O/A alone.

Supplementary Fig. 8

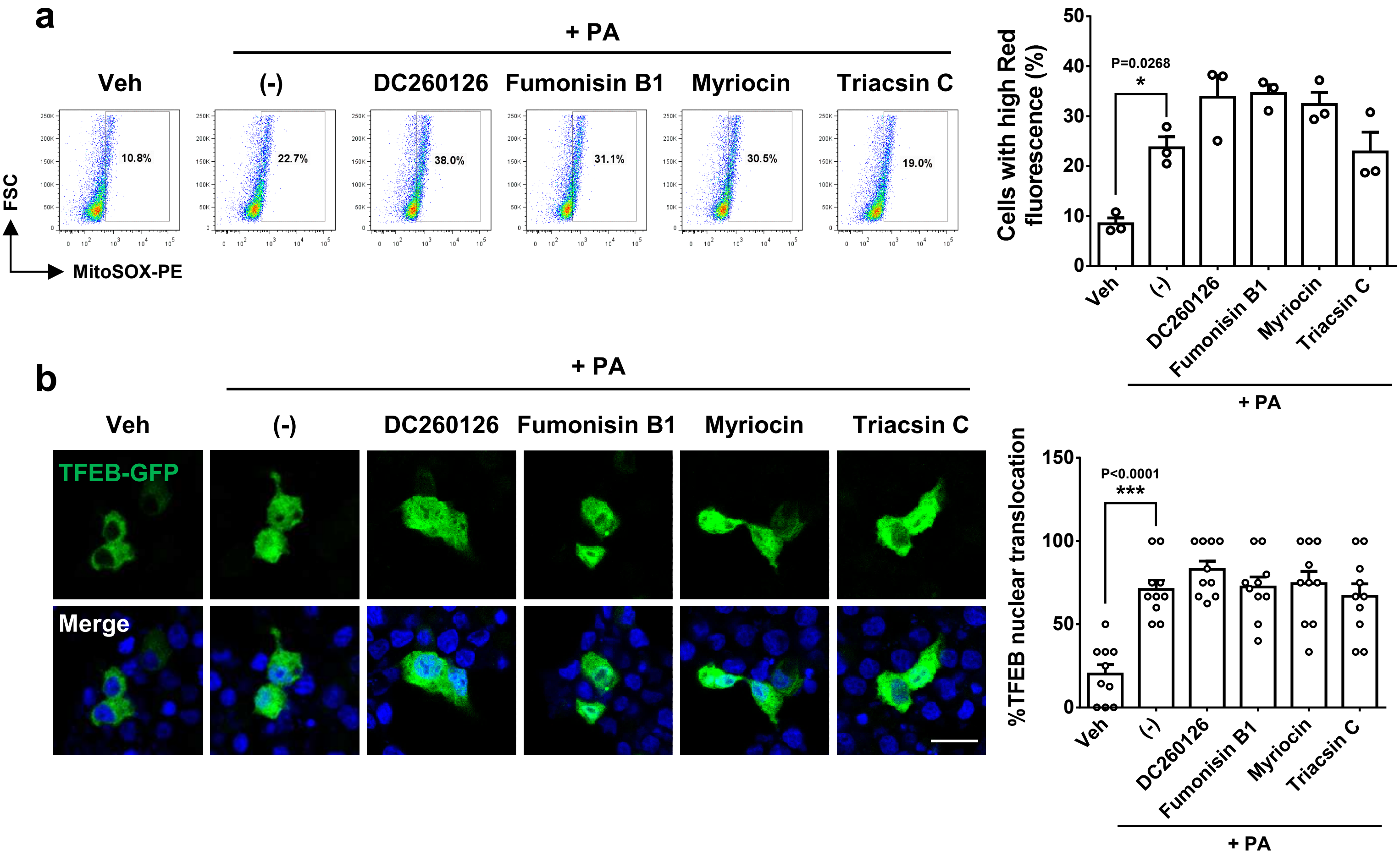

Supplementary Fig. 8. Effect of an antagonist of fatty acid receptors belonging to GPCR family or inhibitors of PA metabolism on mitochondrial ROS and TFEB activation. a After treatment of INS-1 cells with PA for 4 h in the presence or absence of DC260126 (a GPCR antagonist), Triacsin C (an acyl-CoA synthetase inhibitor), Fumonisin B1 (a sphingosine *N*-acyltransferase inhibitor) or Myriocin (a serine palmitoyltransferase inhibitor), mitochondrial ROS accumulation was determined using MitoSOX (right). Representative scattergrams are presented. (left) (*n*=3) **b** After the same treatment of *TFEB-GFP*-transfected INS-1 cells as in (**a**), TFEB nuclear translocation was determined by confocal microscopy (right). Representative fluorescent images are shown (left). (scale bar, 20 μm) (*n*=10) All data in this figure are the means ± SEM from more than 3 independent experiments. *P* values were determined using one-way ANOVA with Tukey's test.

Supplementary Fig. 9

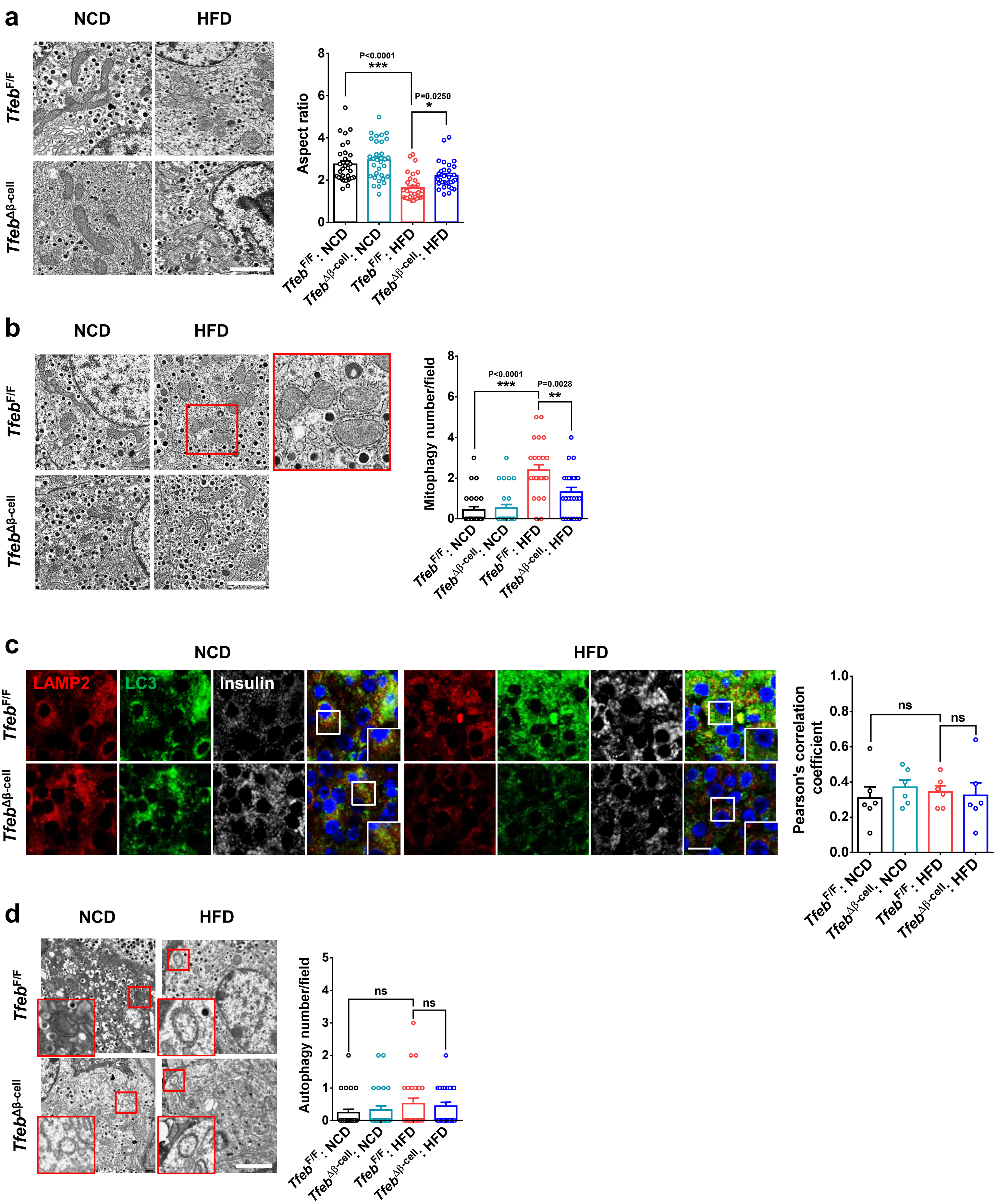

Supplementary Fig. 9. Effect of HFD and β-cell-specific *Tfeb* KO on mitophagy and autophagy. a Electron microscopy (EM) of pancreatic islets from *Tfeb*$^{\Delta\beta\text{-cell}}$ and *Tfeb*$^{F/F}$ mice fed NCD or HFD for 12 weeks (left). Aspect ratio of mitochondria was calculated (right). (scale bar, 2,000 nm) (*n*=31) **b** Representative EM pictures showing autophagosomes surrounding mitochondria in pancreatic islets of mice in (**a**). Rectangle was magnified. (scale bar, 2,000 nm) (*n*=25) **c** Representative pictures of the colocalization of LAMP2 with LC3 in pancreatic islets of *Tfeb*$^{\Delta\beta\text{-cell}}$ and *Tfeb*$^{F/F}$ mice fed NCD or HFD for 12 weeks (left). Pearson's correlation coefficient was calculated (right). Rectangles were magnified. (scale bar, 10 μm) (*n*=6) **d** Representative EM pictures showing autophagosomes without identifiable organelle structure in pancreatic islets of mice in (**a**). Rectangle was magnified. (*n*=25) (scale bar, 2,000 nm) All data in this figure are the means ± SEM from more than 3 independent experiments. *P* values were determined using one-way ANOVA with Tukey's test; ns, not significant.

Supplementary Fig. 10

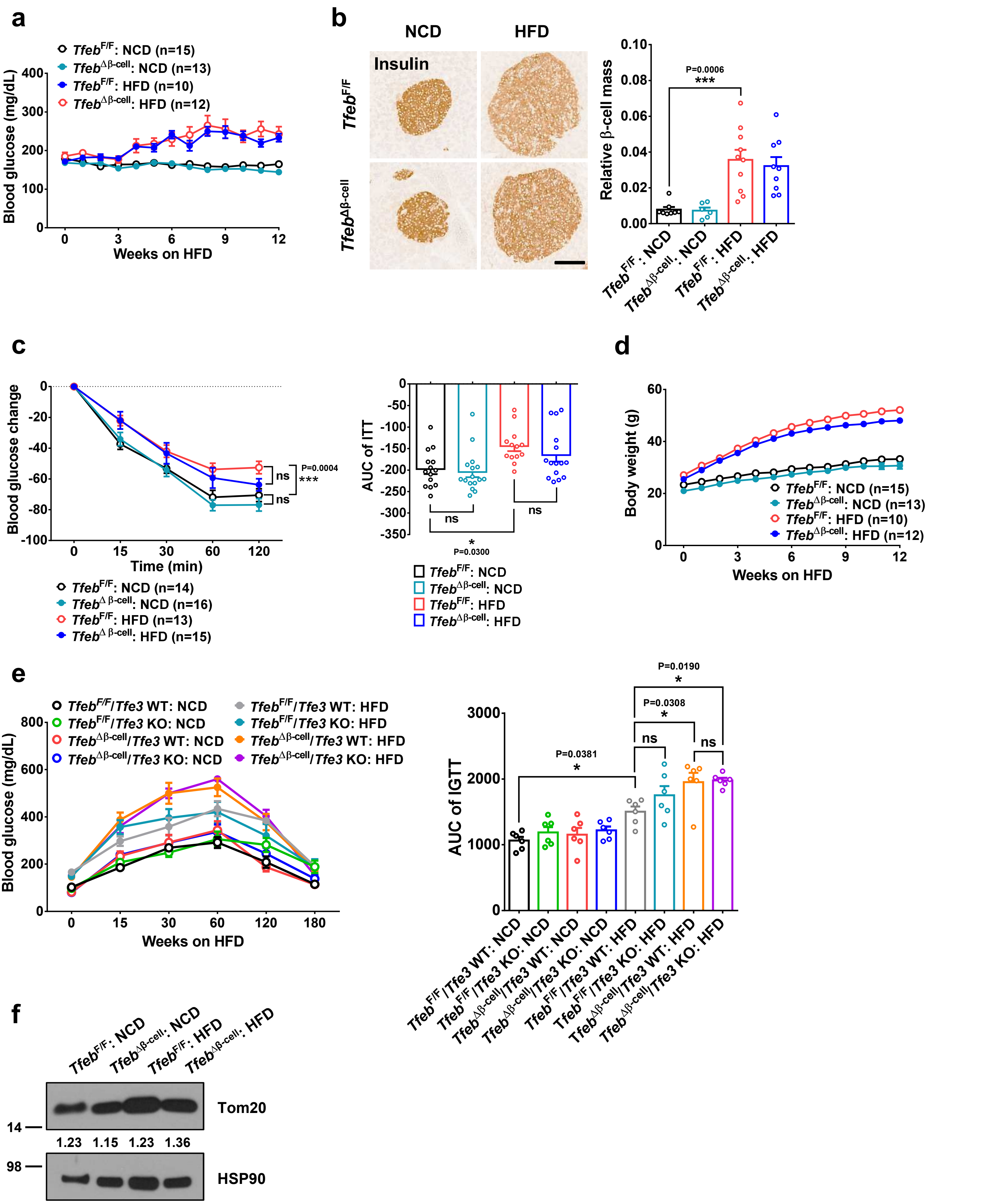

Supplementary Fig. 10. Effect of β-cell-specific *Tfeb* KO on metabolic profile. a Non-fasting blood glucose levels were monitored in *Tfeb*$^{\Delta\beta\text{-cell}}$ and *Tfeb*$^{F/F}$ mice fed NCD or HFD for 12 weeks. **b** Representative insulin immunohistochemistry (left) and β-cell mass (right) of pancreatic islets in *Tfeb*$^{\Delta\beta\text{-cell}}$ and *Tfeb*$^{F/F}$ mice fed NCD or HFD for 12 weeks. (scale bar, 200 μm) (*n*=6 for *Tfeb* $^{\Delta\beta\text{-cell}}$:NCD; *n*=8 for *Tfeb*$^{F/F}$:NCD; *n*=9 for *Tfeb* $^{\Delta\beta\text{-cell}}$:HFD; *n*=10 for *Tfeb*$^{F/F}$:HFD) **c** Insulin tolerance test (ITT) was performed in *Tfeb*$^{\Delta\beta\text{-cell}}$ and *Tfeb*$^{F/F}$ mice fed NCD or HFD for 12 weeks (left). Area under the curve (AUC) was calculated (right). **d** Body weight was monitored in *Tfeb*$^{\Delta\beta\text{-cell}}$ and *Tfeb*$^{F/F}$ mice fed NCD or HFD for 12 weeks. **e** GTT was conducted in *Tfeb*$^{F/F}$/*Tfe3*-WT, *Tfeb*$^{F/F}$/*Tfe3*-KO, *Tfeb*$^{\Delta\beta\text{-cell}}$/*Tfe3*-WT and *Tfeb*$^{\Delta\beta\text{-cell}}$/*Tfe3*-KO mice (left), and AUC was calculated (right). (*n*=6) **f** Immunoblot analysis of pancreatic islets from *Tfeb*$^{\Delta\beta\text{-cell}}$ and *Tfeb*$^{F/F}$ mice fed NCD or HFD for 12 weeks using the indicated Abs. Densitometry of immunoblot bands was performed using ImageJ. Numbers below immunoblot bands indicate the densitometric values normalized to that of the HSP90 band. All data in this figure are the means ± SEM from more than 3 independent experiments. *P* values were determined using one-way ANOVA with Tukey’s test [**b,c,e**] or two-way ANOVA with Bonferroni’s test [**c,e**]; ns, not significant.

Supplementary Fig. 11

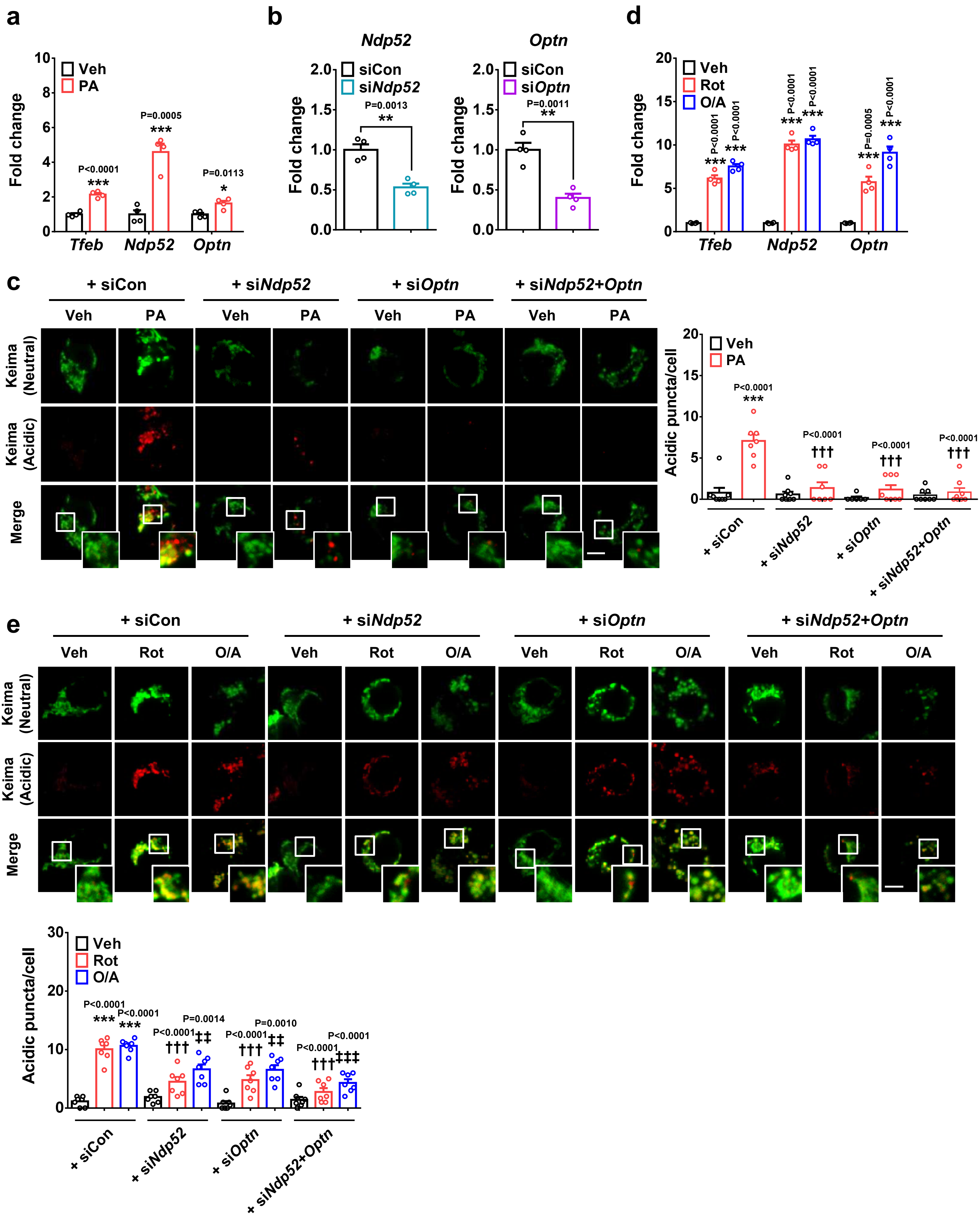

Supplementary Fig. 11. Role of *Ndp52* and *Optn* in mitophagy induced by metabolic or mitochondrial stressors. a After treatment of INS-1 cells with PA for 6 h, expression of the indicated genes was examined by real-time RT-PCR. (*n*=4) **b** Expression of *Ndp52* and *Optn* mRNA in INS-1 cells transfected with *Ndp52* or *Optn* siRNA was examined by real-time RT-PCR. (*n*=4) **c** INS-1 cells transfected with *Ndp52* or *Optn* siRNA together with *pMito-Keima* were treated with PA for 18 h, and the numbers of red puncta indicating the occurrence of mitophagy were counted (right). Representative fluorescent images are presented (left). (scale bar, 5 μm) (*n*=6 for si*Optn*; *n*=7 for PA or si*Ndp52*+PA; *n*=8 for SiCon, si*Ndp52*, si*Optn*+PA, si*Ndp52*+*Optn* or si*Ndp52*+*Optn*+PA) (SiCon, control siRNA) **d** After treatment of INS-1 cells with mitochondrial stressors for 6 h, expression of the indicated genes was examined by real-time RT-PCR. (n=4) **e** INS-1 cells transfected with *Ndp52* or *Optn* siRNA together with *pMito-Keima* were treated with rotenone or O/A for 18 h, and the numbers of red puncta indicating the occurrence of mitophagy were counted (lower). Representative fluorescent images are presented (upper). (scale bar, 5 μm) (*n*=7) (SiCon, control siRNA) Rectangles were magnified. All data in this figure are the means ± SEM from more than 3 independent experiments. *P* values were determined using one-way ANOVA with Tukey's test. *, compared to Veh-treated cells; †, compared to cells treated with Rot alone; ‡, compared to cells treated with O/A alone.

Supplementary Fig. 12

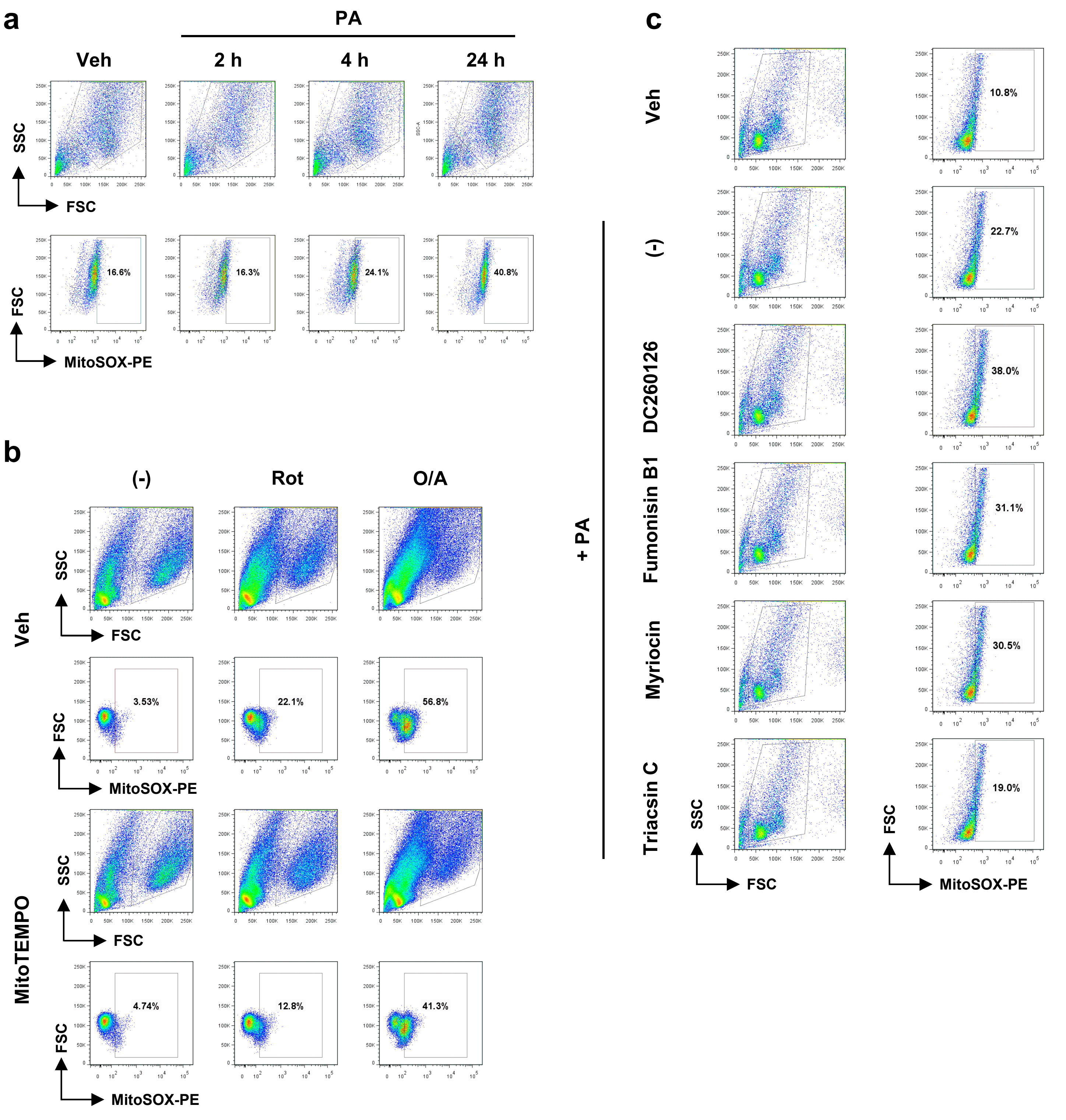

Supplementary Fig. 12. Gating strategy for flow cytometry. a Gating strategy and representative scattergrams for Fig. 4a. **b** Gating strategy and representative scattergrams for Supplementary Fig. 5c. **c** Gating strategy and representative scattergrams for Supplementary Fig. 8a.

Supplementary Fig. 1. a

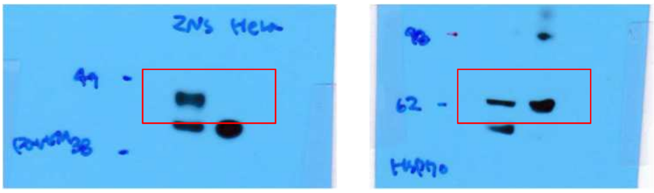

Parkin HSP70

Supplementary Fig. 1. b

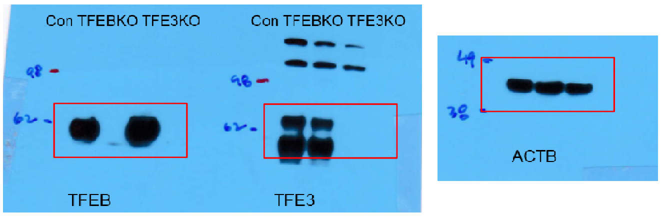

Supplementary Fig. 3. c

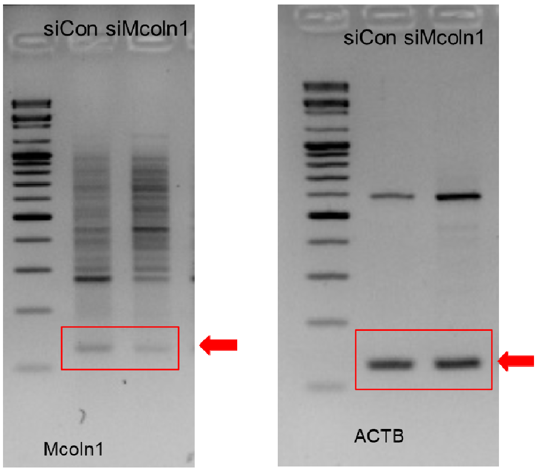

Supplementary Fig. 4. a

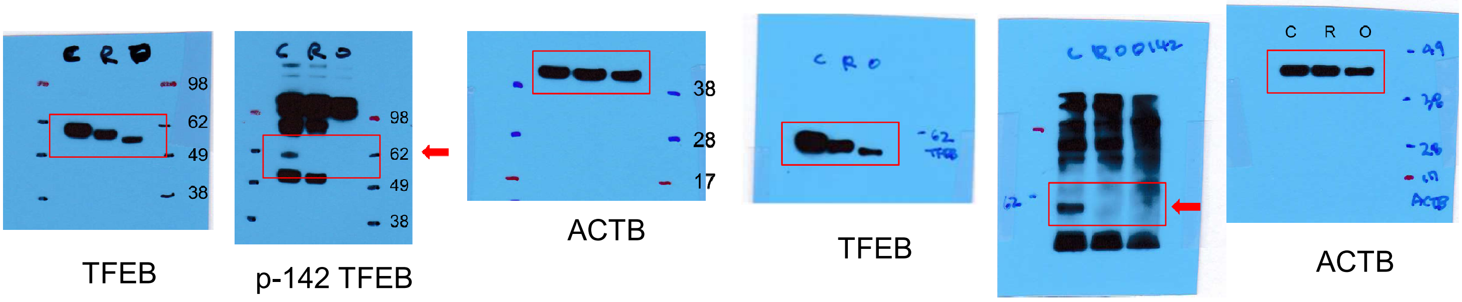

TFEB p-142 TFEB ACTB TFEB p-142 TFEB ACTB

Supplementary Fig. 4. b

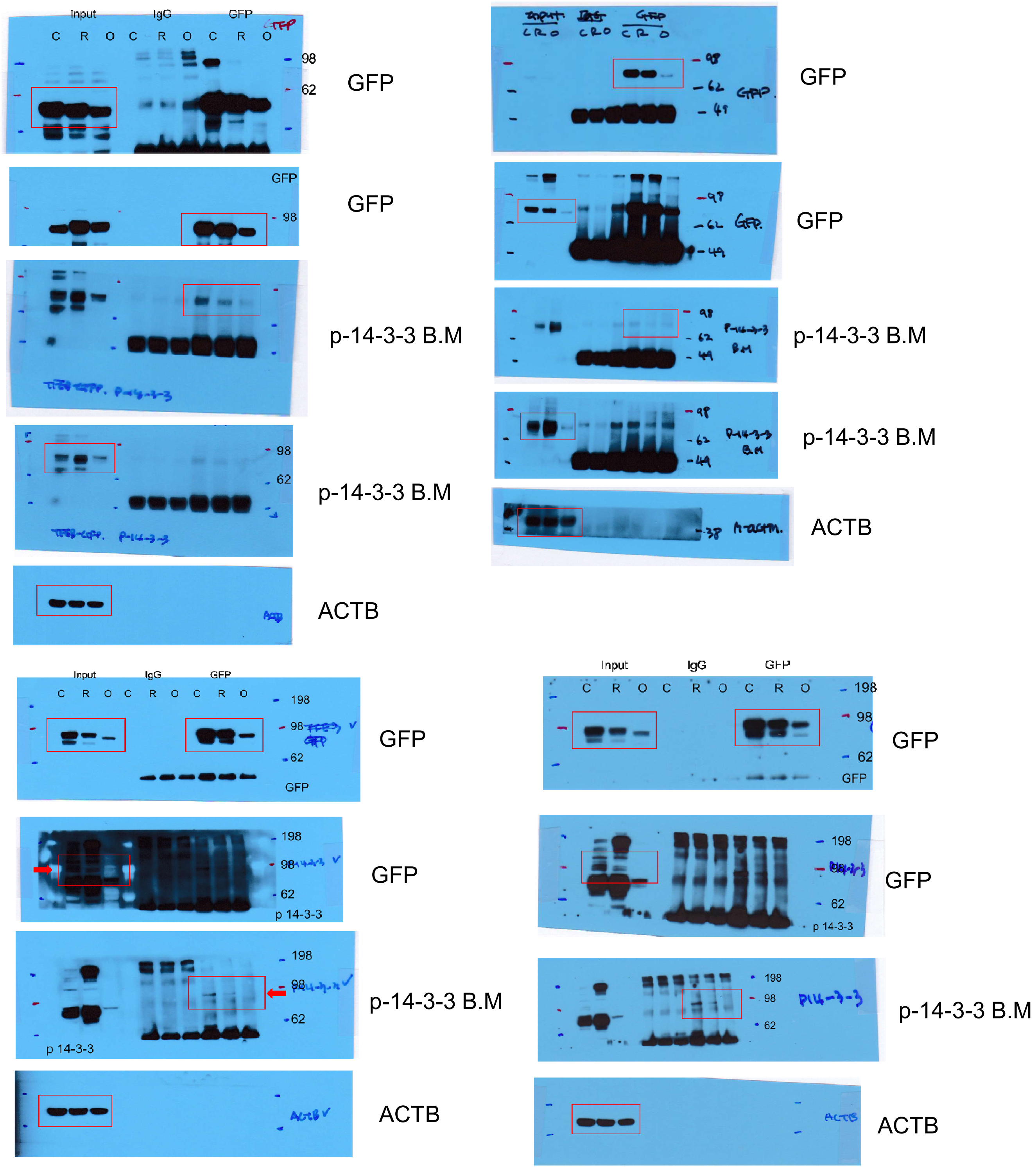

Supplementary Fig. 10. f

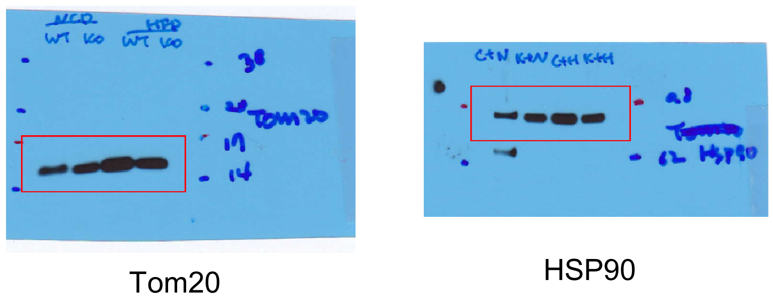

Supplementary Table 1. **Primer sequences of TFEB target genes (human) for real-time RT-PCR**

Name	Forward (5'→3')	Reverse (5'→3')
TFEB	CGCATCAAGGAGTTGGGAAT	GAGCTGCTTGTTGGTCATCT
NDP52	TCACCCAGCATTTCATCCCTC	GTCCTTGGCTCCTCCATTTG
OPTN	GAGAAGGCTCTGGCTTCCAA	GTCATGGTTTCCAGGTCCTCTT
GAPDH	TGCACCACCAACTGCTTAGC	GGCATGGACTGTGGTCATGAG

Supplementary Table 2. Primer sequences of TFEB target genes (mouse) for real-time RT-PCR

Name	Forward (5'→3')	Reverse (5'→3')
Parkin	AAACCCACCTACAACAGCTT	GGTGAGGGTTGCTTGTTTGC
Tfeb	GTCATTGACAACATTATGCGCC	GCGTGTTAGGCATCTTGCATCT
Tfe3	CCGTGTTCCTGCTATTGGAA	CGTAGAAGCTGTCAGGATCG
Map1lc3b	AGATCCCAGTGATTATAGAGCGA	AGGCTTGGTTAGCATTGAGC
Sqstm1	AGATGCCAGAATCGGAAGGG	GGAGAGGGACTCAATCAGCC
Lamp1	CCTACGAGACTGCGAATGGT	CCACAAGAACTGCCATTTTTC
Clcn7	GAAAGTGTCTTGGTCCGGC	GGTGTGAGGAGGATCGACTT
Becn1	AGGCTGAGGCGGAGAGATT	TCCACACTCTTGAGTTCGTCAT
Uvrag	CAAGCTGACAGAAAAGGAGCGAG	GGAAGAGTTTGCCTCAAGTCTGG
Mcoln1	GCGCCTATGACACCATCAA	TATCCTGGCACTGCTCGAT
Ctsa	GAACGACCCAAAGAACAGCC	CACCATCTGGCTGGATCAGA
Ctsd	AGTGGCTTCATGGGAATGGA	GCAAAGCCGACCCTATTGTT
Ctsf	AGGCCTGGAACTTGTCGTC	ATTGCAAGGTGGCTCCTCTA
Atp6v0e1	GATCGTGATGAGCGTGTTCT	GGTCCAAACAGAGGATTGAGC
Atp6v1h	CGAGGACAAGCAAGAGATGC	CCATGGTTAGTATGTACTGCACTG
Ndp52	TGGCAACTTCTCTCAGGTCCTGTT	TCCTTGCGTCGAGGGATGAACTTT
Optn	AGGGAGGCAGTAGACAGTCC	CACTTGGGGCAGGAGTGAAT
Nbr1	CCCCAGATTGGTTTACAAGC	TCCACCGTTTCCTTAACCAC
Tbk1	TATCTTTGTCACGAGCCGGG	CCGTACCCCCTTTCGCATTA
Tax1bp1	GGCCCTGACCGATTCACAA	TGGCACTCCAAGTGTGCATTA

Name	Forward (5’→3’)	Reverse (5’→3’)
Tfam	AGATATGGGTGTGGCCCTTG	AAAGCCTGGCAGCTTCTTTG
PGC-1α	AGCCGTGACCAGTGACAACGAG	GCTGCATGGTTCTGAGTGCTAAG
ESRRα	AGAGACTGAGACTGAACCCC	TTTGGGTAGAGAGCTGAGCA
NRF1	CAGACACGTTTGCTTCGGAAA	CCCACTCGCGTCGTGTACT
NRF2	GATCAGGCGACATGTTAACGTT	AGAGCCCAGTCAAACCCTTTC
COX1	TGCTAGCCGCAGGCATTACT	CGGGATCAAAGAAAGTTGTGTTT
COX2	CAGGCCGACTAAATCAAGCAA	GAGCATTGGCCATAGAATAATCCT
S18	AGGTTCTGGCCAACGGTCTAG	CCCTCTATGGGCTCGAATTTT

Supplementary Table 3. Primer sequences of TFEB target genes (rat) for real-time RT-PCR

Name	Forward (5’→3’)	Reverse (5’→3’)
Mcoln1	TCTCCAGACACGGAGACAAC	AACTCGTTCTGCAGCAGGAAGC
Actb	TAAGGCCAACCGTGAAAAGA	ATCACAATGCCAGTGGTACG
Tfeb	GGTCTTGGGCAAATCCCTTC	ATCCTCGGAGTCTTTAAGCG
Ndp52	CATGCCCGATGACCTAAACA	CCCTGGACCAAACCATCTTC
Optn	GGGTTTCCCAGAACCGACTT	AAGGTCCGCTTTCTCAAGCC
Rpl32	GCTGCTGATGTGCAACAAAT	TTCATTCTCTTCGCTGCGT

Supplementary Table 4. Sequence of primers used for ChIP assay

Name	Forward (5’→3’)	Reverse (5’→3’)
NDP52	CTGAGCCCAGCCTCTTTTTTTTTT	CCGTAGTGGCTTACGCCTGTTATC
OPTN	AAACAAAGGAGTTACTGACTTTTC	TAGCTTCCATAATTCCCGTGTGTT
ACTB	ATGCAGCGATCAGTGGCGT	TCCAGCTTCTTGTCACCACCTC
