# Supplementary material for: Lysosomal Ca2+-mediated TFEB activation modulates mitophagy and functional adaptation of pancreatic β-cells to metabolic stress
Source: Nat Commun. 2022 Mar 14;13:1300. doi: 10.1038/s41467-022-28874-9 (PMC8921223; doi:10.1038/s41467-022-28874-9)
Supplement: Supplementary file 3 — Description of Additional Supplementary Information [file 41467_2022_28874_MOESM3_ESM.docx]

Supplementary Information

Peer Review File

Reporting Summary
