# Supplementary material for: Lysosomal Ca2+-mediated TFEB activation modulates mitophagy and functional adaptation of pancreatic β-cells to metabolic stress
Source: Nat Commun. 2022 Mar 14;13:1300. doi: 10.1038/s41467-022-28874-9 (PMC8921223; doi:10.1038/s41467-022-28874-9)
Supplement: Supplementary file 4 — Reporting Summary [file 41467_2022_28874_MOESM4_ESM.pdf]

nature portfolio

Corresponding author(s): Myung-Shik Lee
Last updated by author(s): Dec 22, 2021

Reporting Summary

Nature Portfolio wishes to improve the reproducibility of the work that we publish. This form provides structure for consistency and transparency in reporting. For further information on Nature Portfolio policies, see our Editorial Policies and the Editorial Policy Checklist.

Statistics

For all statistical analyses, confirm that the following items are present in the figure legend, table legend, main text, or Methods section.

n/a	Confirmed	
☐	☒	The exact sample size (*n*) for each experimental group/condition, given as a discrete number and unit of measurement
☐	☒	A statement on whether measurements were taken from distinct samples or whether the same sample was measured repeatedly
☐	☒	The statistical test(s) used AND whether they are one- or two-sided<br>*Only common tests should be described solely by name; describe more complex techniques in the Methods section.*
☒	☐	A description of all covariates tested
☐	☒	A description of any assumptions or corrections, such as tests of normality and adjustment for multiple comparisons
☐	☒	A full description of the statistical parameters including central tendency (e.g. means) or other basic estimates (e.g. regression coefficient) AND variation (e.g. standard deviation) or associated estimates of uncertainty (e.g. confidence intervals)
☐	☒	For null hypothesis testing, the test statistic (e.g. *F*, *t*, *r*) with confidence intervals, effect sizes, degrees of freedom and *P* value noted<br>*Give P values as exact values whenever suitable.*
☒	☐	For Bayesian analysis, information on the choice of priors and Markov chain Monte Carlo settings
☒	☐	For hierarchical and complex designs, identification of the appropriate level for tests and full reporting of outcomes
☐	☒	Estimates of effect sizes (e.g. Cohen's *d*, Pearson's *r*), indicating how they were calculated

Our web collection on statistics for biologists contains articles on many of the points above.

Software and code

Policy information about availability of computer code

Data collection	GraphPad Prism 6 software, Zen 3.0(blue) software, ImageJ software, Fiji software, WaveTM software, FlowJo software, MetaFluor Software, QuantStudio Design & Analysis software
Data analysis	Statistics and Graphs were performed using GraphPad Prism 6 software (Ver. 6.01).<br>Fluorescent confocal images were exported using Zen 3.0 software (Ver. 3.0, blue).<br>Band intensity of immunoblot and mean pixel intensity of fluorescence per cell were quantified using ImageJ software (Ver. 1.46r).<br>mean pixel intensity of DAB per cell was quantified using Fiji software (Ver. Life-Line).<br>Analysis of gene expression level was run and exported using QuantStudio Design & Analysis software (Ver. 1.5.1).<br>Oxygen consumption was analyzed using WaveTM software (Ver. 2.3.0.19).<br>Fura-2 was analyzed using MetaFluor Software.<br>Fluorescence of MitoSox-Red was analyzed using BD FASCVerse flow cytometry system (BD FACSuiteTM software) and FlowJo software (Ver. 7.6.5).

For manuscripts utilizing custom algorithms or software that are central to the research but not yet described in published literature, software must be made available to editors and reviewers. We strongly encourage code deposition in a community repository (e.g. GitHub). See the Nature Portfolio guidelines for submitting code & software for further information.

Data

Policy information about availability of data

All manuscripts must include a data availability statement. This statement should provide the following information, where applicable:

- Accession codes, unique identifiers, or web links for publicly available datasets
- A description of any restrictions on data availability
- For clinical datasets or third party data, please ensure that the statement adheres to our policy

The data sets generated in the current study are available from the corresponding author on reasonable request. Source data generated for figures in this study are provided in the Source Data file.

Field-specific reporting

Please select the one below that is the best fit for your research. If you are not sure, read the appropriate sections before making your selection.

☒ Life sciences ☐ Behavioural & social sciences ☐ Ecological, evolutionary & environmental sciences

For a reference copy of the document with all sections, see nature.com/documents/nr-reporting-summary-flat.pdf

Life sciences study design

All studies must disclose on these points even when the disclosure is negative.

Sample size	For animal experiments, the sample size was determined based on the similar previous studies conducted using similar methodologies in our laboratory. Detailed sample size was described in the figure legends. In experiments other than animal experiment, sample sizes were chosen to yield statistically significant differences based on our preliminary data and published papers by other researchers.
Data exclusions	The samples for both in vivo and in vitro experiments were not excluded from all the analyses.
Replication	All findings of this study were reproducible reliably. All data in this manuscript are from at least more than 3 independent experiments.
Randomization	For animal experiments, age-matched male mice were randomly assigned to experimental groups. Samples were prepared, treated, processed and analysed in a random order.
Blinding	The investigators were not blinded to allocation during experiments and outcome assessment. However, to minimize any potential bias, all male mice were randomly assigned to experimental groups or control groups (age-matched). All samples were collected and analyzed under the same conditions.

Reporting for specific materials, systems and methods

We require information from authors about some types of materials, experimental systems and methods used in many studies. Here, indicate whether each material, system or method listed is relevant to your study. If you are not sure if a list item applies to your research, read the appropriate section before selecting a response.

Materials & experimental systems

n/a	Involved in the study	
☐	☒	Antibodies
☐	☒	Eukaryotic cell lines
☒	☐	Palaeontology and archaeology
☐	☒	Animals and other organisms
☒	☐	Human research participants
☒	☐	Clinical data
☒	☐	Dual use research of concern

Methods

n/a	Involved in the study	
☒	☐	ChIP-seq
☐	☒	Flow cytometry
☒	☐	MRI-based neuroimaging

Antibodies

Antibodies used

The following antibodies were used for immunoblotting (IB), immunoprecipitation (IP), Immunofluorescence (IF), immunohistochemistry (IHC), Chromatin Immunoprecipitation (ChIP), Proximity ligation assay (PLA) :

1. TFEB (Bethyl Laboratory, A303-673A, Lot. 6, IF 1:200, IP 1:1,000, IB 1:1,000 dilution),
2. TFE3 (Sigma-Aldrich, HPA023881, Lot. G114961, IF 1:200, IP 1:1,000, IB 1:1,000 dilution),
3. GFP (AbFrontier, LF-PA0043, Lot. OJA01-01, IP 1:1,000, IB 1:1,000, ChIP 1:1,000 dilution),
4. TOM20 (Cell Signaling Technology, 42406S, clone: D8T4N, IF 1:200, IHC 1:200, IB 1:1,000 dilution),

5. HA (Cell Signaling Technology, 2367S, clone: 6E2, Lot. 5, IF 1:200 dilution),
6. ORP1L (Abcam, ab203352, Lot. GR246475-4, PLA 1:200 dilution),
7. VAPA (Santa Cruz Biotechnology, sc-293278, clone: 4C12, Lot. F2018, PLA 1:200 dilution),
8. LAMP2 (Abcam, ab13524, clone: GL2A7, IHC 1:200 dilution),
9. Flag (Sigma, F7425, Lot. 086M4803V, IF 1:200 dilution),
10. LC3 (MBL, M152-3, clone: 4E12, Lot. 056, IHC 1:200 dilution),
11. 4 Hydroxynonenal (Abcam, ab46545, IHC 1:200 dilution),
12. Parkin (Santa Cruz Biotechnology, sc-32282, clone: PRK8, Lot. L1216, IB 1:1,000 dilution),
13. phospho TFEB (Ser142) (Merk Millipore, ABE1971, Lot. 3602264, IB 1:1,000 dilution),
14. Phospho-(Ser) 14-3-3 Binding Motif (Cell Signaling Technology, 9601S, Lot. 13, IB 1:1,000 dilution),
15. beta-Actin (C4) (ACTB; Santa Cruz Biotechnology, sc-47778, clone: C4, Lot. L1620, IB 1:1,000 dilution),
16. HSP 70 (F-3) (Santa Cruz Biotechnology, sc-373867, clone: AC88, Lot. K0416, IB 1:1,000 dilution),
17. Insulin (Cell Signaling Technology, 4590s, Lot. 2, IHC 1:200 dilution),
18. insulin (Invitrogen, PA1-26938, Lot. WC3199382, IHC 1:200 dilution),
19. HSP90 (Santa Cruz Biotechnology, sc-13119, clone: F-8, Lot. J1920, IB 1:1,000 dilution),
20. Alexa 488-anti-rabbit IgG (H+L) (Invitrogen, A11008, IF 1:200, IHC 1:200 dilution),
21. Alexa 594-anti-mouse IgG (H+L) (Invitrogen, A11005, IF 1:200, IHC 1:200 dilution),
22. Alexa 594-anti-rat IgG (H+L), (Invitrogen, A11007, IHC 1:200 dilution),
23. Alexa 647-anti-guinea pig IgG (H+L), (Invitrogen, A21450, IHC 1:200 dilution),
24. Alexa 568-anti-rabbit IgG (H+L) (Invitrogen, A11011, IHC 1:200 dilution),
25. Alexa 405-anti-rabbit IgG (H+L) (Abcam, ab175651, IF 1:200 dilution),
26. Alexa 488-anti-mouse IgG (H+L) (Invitrogen, A11001, IF 1:200, IHC 1:200 dilution),
27. Biotinylated anti-rabbit IgG (H+L) (Vector, BA-1000, IHC 1:1,000 dilution)
28. Horseradish peroxidase-conjugated anti-rabbit IgG (Cell Signaling Technology, 7074S, IB 1:1,000 dilution)

Validation

The information above can also be found in the Methods section. For all antibodies, additional information on application, species cross-reactivity or related publications can be found on the manufacturer's website.

(Applications / Species Cross-Reactivity / Source)
1. TFEB (Bethyl Laboratory, A303-673A): IB (1:2,000-1:10,000), IP (2 to 10"g per mg lysate), IHC-P (1:500-1:2,000), IF (1:250-1:1,000) / Human, Mouse / Rabbit
2. TFE3 (Sigma Aldrich, HPA023881): IB (1:100-1:250), IF (1-4"g per mL), IHC (1:500-1:1,000) / Human, Mouse, Rat / Rabbit
3. GFP (AbFrontier LF-PA0043, IP 1:1,000): IB (1:1,000-5,000), IP (1-2"l) / - / Rabbit
4. TOM20 (Cell Signaling Technology, 42406S): IB (1:1,000), IF (1:100-1:400), IHC-P (1:100-1:400)/ Human, Mouse, Rat, Monkey/ Rabbit
5. HA (Cell Signaling Technology, 2367S): IF (1:100)/ All/ Mouse
6. ORP1L (Abcam, ab203352): IHC-P (1:100-1:500)/ Mouse, Rat/ Rabbit
7. VAPA (Santa Cruz Biotechnology, sc-293278): IB (1:100-1:1000), IF (1:50-1:500), IHC-P (1:50-1:500)/ Human, Mouse, Rat/ Mouse
8. LAMP2 (Abcam, ab13524): IHC (1:500), IF (1:100-1:500)/ Mouse, Rabbit, Human/ Rat
9. Flag (Sigma, F7425): IB (1.0-2.5 μg/ml), IF (5-10 μg/ml)/ - /Rabbit
10. LC3 (MBL, M152-3): IF (40 μg/ml), IHC (1:50)/ Human, Mouse, Rat, Hamster/ Mouse
11. 4 Hydroxynonenal (Abcam, ab46545): IB (1:500), ELISA (1:4,000-1:20,000)/ Species independent/ Rabbit
12. Parkin (Santa Cruz Biotechnology, sc-32282): IB (1:100-1:1000), IF (1:50-1:500), IHC-P (1:50-1:500)/ Human, Mouse, Rat/ Mouse
13. phospho TFEB (Ser142) (Merk Millipore, ABE1971): IB (1:12,500) / Human (Predicted to react with Mouse, Rat) / Rabbit
14. Phospho-(Ser) 14-3-3 Binding Motif (Cell Signaling Technology, #9601): IB (1:1,000), IP (1:50), IHC (1:50), ELISA (1:1,000) / All / Rabbit
15. beta-Actin (C4) (ACTB; Santa Cruz Biotechnology, SC-47778): IB (1:100-1:1,000), IP (1-2 "g per 100-500 "g of total protein), IF(1:50-1:500), IHC (1:50-1:500), ELISA (1:30-1:3,000) / Mouse, Rat, Human, Avian, Bovine, Canine, Porcine, Rabbit / Mouse
16. HSP 70 (F-3) (Santa Cruz Biotechnology, sc-373867): IB (200 μg/ml), IHC-P (200 μg/ml)/ Human, Mouse, Rat/ Mouse
17. Insulin (Cell Signaling Technology, 4590s): IHC-P (1:100), ICC/IF (1:100) / Human, Mouse, Rat / Rabbit
18. insulin (Invitrogen, PA1-26938): IHC-P (1:50-1:100), IHC-F (1:50-1:100), IF (1:50-1:100)/ Human, Mouse/ Guinea pig
19. HSP90 (Santa Cruz Biotechnology, sc-13119): IB (1:100-1:1000), IF (1:50-1:500), IHC-P (1:50-1:500)/ Human, Mouse, Rat/ Mouse

*Immunoblotting (IB), immunoprecipitation (IP), immunofluorescence (IF), immunocytochemistry (ICC), immunohistochemistry (IHC)

Eukaryotic cell lines

Policy information about cell lines

Cell line source(s)

INS-1 insulinoma cells were provided by Dr. C. Wollheim, University of Geneva, Switzerland who originally developed the cell line.
Primary mouse islet cells were isolated from TfebF/F, TfebΔβ-cell and Nfe2l2-KO mice.
1.1B4 cells were obtained from the ECACC through Fadzilah Adibah Abdul Majid, Universiti Malaysia Terengganu.
Tfeb- or Tfe3-KO INS-1 cells were generated by using CRISPR/Cas9 technology.
J774A.1 cells were purchased from ATCC.

Authentication

INS-1, 1.1B4 and J774A.1 cells were not authenticated but displayed expected morphology.
Tfeb- or Tfe3-KO INS-1 cells were authenticated by immunobloting using antibodies against TFEB, TFE3.

Mycoplasma contamination

All cells were negative for mycoplasma contamination, except primary mouse islet cells. Primary islet cells were not tested for mycoplasma contamination.

Commonly misidentified lines
(See ICLAC register)

No commonly misidentified cell lines were used.

Animals and other organisms

Policy information about studies involving animals; ARRIVE guidelines recommended for reporting animal research

Laboratory animals	Rip-Cre mice were purchased from Jackson Laboratory.<br>TfebF/F mice were generated by breeding Tfebtm1a(EUCOMM)Wtsi mice (Mutant Mouse Resource and Research Center, MMRRC) with FLPeR mice (Wellcome Trust Sanger Institute).<br>Tfe3-KO mice were purchased from Jackson Laboratory.<br>Nfe2l2-KO mice were obtained from Yamamoto M.<br>As a diet-induced diabetic model, 8-week-old mice were fed HFD (60% kcal fat, Research Diet #D12492) for 8 weeks. Male mice with age range 12 weeks were used in all experiments.
Wild animals	This study did not involve wild animals.
Field-collected samples	This study did not involve field collected samples.
Ethics oversight	All Mouse experiments were approved by the IACUC of the Department of Laboratory Animal Resources of Yonsei University College of Medicine, an AAALAC-accredited unit.

Note that full information on the approval of the study protocol must also be provided in the manuscript.

Flow Cytometry

Plots

Confirm that:

☒ The axis labels state the marker and fluorochrome used (e.g. CD4-FITC).

☒ The axis scales are clearly visible. Include numbers along axes only for bottom left plot of group (a 'group' is an analysis of identical markers).

☒ All plots are contour plots with outliers or pseudocolor plots.

☒ A numerical value for number of cells or percentage (with statistics) is provided.

Methodology

Sample preparation	After treatment of INS-1 cells seeded in 12 well plate with rotenone (100 nM) and O/A (200 nM+125 nM) for 1 h, in the presence or absence of MitoTEMPO, cells were incubated with MitoSOX-red (5 μM), an indicator of mitochondrial ROS for 30 min in RPMI-1640 medium.<br>After treatment of INS-1 cells seeded in 12 well plate with PA (400 μM) for 2-24 h, in the presence or absence of of DC260126, Triacsin C, Fumonisin B1 or Myriocin, cells were incubated with MitoSOX-red (5 μM), an indicator of mitochondrial ROS for 30 min in RPMI-1640 medium.
Instrument	Fluorescence of MitoSOX-Red was analyzed by flow cytometry (FACSVerse, BD Biosciences)
Software	FlowJo software
Cell population abundance	More than 90% of cells were identified as live and single cells. More than 10,000 cells were identified as stained cells.
Gating strategy	Live cells were gated with FCS-A/SSC-A (x-axis/y-axis) followed PE-A/FCS-A (x-axis/y-axis) and subsequently gated as indicated in the figures.

☒ Tick this box to confirm that a figure exemplifying the gating strategy is provided in the Supplementary Information.
